# Rates of cervical screening amongst females admitted to the psychiatric inpatient hospital in Jersey, Channel Islands

**DOI:** 10.1192/bjo.2021.609

**Published:** 2021-06-18

**Authors:** Jade Wright

**Affiliations:** St Saviours Hospital, Jersey

## Abstract

**Aims:**

Patients with enduring mental health conditions are known to have higher morbidity and mortality rates than the general population. It has been identified that this is due to lifestyle risk factors, medication side effects and barriers to receiving physical health care. National screening programmes; including cervical screening, save lives, however depends upon patient engagement. We hypothesised that due to the factors stated above, psychiatric inpatients are more at risk of cervical cancer and less likely to engage in cervical screening. This study aimed to assess the cervical screening history of patients discharged from the psychiatric inpatient hospital in Jersey, Channel Islands.

**Method:**

Using computerised laboratory records, the cervical smear history of female patients discharged from the paychiatric inpatient hospital was analysed. Inclusion criteria were: being aged between 25–64 years and having a cervix in situ. Exclusion criteria were total hysterectomy. Cervical smear history was compared to the national guidelines of having routine smears every 3 years for women aged 25–49 and every 5 years for women aged between 50–64 years.

**Result:**

In the period 1 December 2019–1 December 2020 there were 45 females discharged from the psychiatric inpatient hospital that fit the inclusion criteria. 26 (58%) were up to date with their cervical smears in accordance with national guidelines. 12 (27%) had previously had a smear but were not up to date. 19 smears were done at the GP, 13 at the sexual health clinic and 6 at gynaecology clinic. 7 (16%) had never had a cervical smear. Of these 7 patients it was identified that one patient was in a same sex relationship and one was a victim of sexual assault.

**Conclusion:**

58% of women discharged from the psychiatric inpatient hospital were up to date with their smears. This is down from the 72.2% coverage rate of the general population. Although this was a small study, it highlights that engagement with cervical screening amongst psychiatric inpatients is less than the general population. Admission presents a crucial contact between patients and healthcare services and this could be utilised to engage patients in physical health screening. Cervical screening history could be checked upon admission and patients not adequately screened, assisted to make an appointment on discharge.

